# Sequencing of a Wild Apple (*Malus baccata*) Genome Unravels the Differences Between Cultivated and Wild Apple Species Regarding Disease Resistance and Cold Tolerance

**DOI:** 10.1534/g3.119.400245

**Published:** 2019-05-24

**Authors:** Xilong Chen, Shiming Li, Dong Zhang, Mingyu Han, Xin Jin, Caipin Zhao, Songbo Wang, Libo Xing, Juanjuan Ma, Jingjing Ji, Na An

**Affiliations:** * College of Horticulture, Northwest A&F University, Yangling 712100, Shannxi, China; ‡ College of Life Sciences, Northwest A&F University, Yangling 712100, Shannxi, China; † BGI Genomics, BGI-Shenzhen, Shenzhen 518083, China

**Keywords:** Siberian crab apple (*Malus baccata*), genome sequencing, resistance gene analogs (RGAs), cold-related genes

## Abstract

*Malus baccata* is one of four wild apple species that can hybridize with the cultivated apple species (*Malus domestica*). It is widely used in high-latitude apple-producing areas as a rootstock and breeding resource because of its disease resistance, and cold tolerance. A lack of a reference genome has limited the application of *M. baccata* for apple breeding. We present a draft reference genome for *M. baccata*. The assembled sequence consisting of 665 Mb, with a scaffold N50 value of 452 kb, included transposable elements (413 Mb) and 46,114 high-quality protein-coding genes. According to a genetic map derived from 390 sibling lines, 72% of the assembly and 85% of the putative genes were anchored to 17 linkage groups. Many of the *M. baccata* genes under positive selection pressure were associated with plant–pathogen interaction pathways. We identified 2,345 Transcription factor-encoding genes in 58 families in the *M. baccata* genome. Genes related to disease defense and cold tolerance were also identified. A total of 462 putative nucleotide-binding site (NBS)-leucine-rich-repeat (LRR) genes, 177 Receptor-like kinase (RLK) and 51 receptor-like proteins (RLP) genes were identified in this genome assembly. The *M. baccata* genome contained 3978 cold-regulated genes, and 50% of these gene promoter containing DREB motif which can be induced by *CBF* gene. We herein present the first *M. baccata* genome assembly, which may be useful for exploring genetic variations in diverse apple germplasm, and for facilitating marker-assisted breeding of new apple cultivars exhibiting resistance to disease and cold stress.

Apple is one of the most extensively cultivated temperate zone tree fruits, and is popular among consumers worldwide. Global apple production has rapidly increased in recent years, reaching about 89 million tons in 2016 (FAOSTAT: http://www.fao.org/faostat). Apple diseases and frost damage can cause significant decreases in yield. Developing apple varieties exhibiting cold tolerance and pathogen resistance is important for ensuring apple fruits can continue to be produced in countries that experience cold conditions and for overcoming the adverse effects of an increasing variety of diseases and climate change. The resistance to diseases and cold stress varies considerably among different apple species, with wild apple species exhibiting very good disease resistance and cold tolerance (Volk *et al.* 2015). Therefore, incorporating wild apple genetic resources into apple breeding programs to select disease- and cold-resistant varieties may be relevant for sustainable apple production.

Because apple fruits are a perennial crop, breeding new varieties is time consuming and labor intensive (Peace and Norelli 2009). Genome data and marker-assisted selection can greatly accelerate the breeding process (Badenes *et al.* 2016). Many perennial fruit tree genomes have recently been sequenced (Velasco *et al.* 2010; Zhang *et al.* 2012; Wu *et al.* 2013; International Peach Genome Initiative *et al.* 2013; Chagné *et al.* 2014; Li *et al.* 2016). Moreover, genome re-sequencing provides valuable information for facilitating marker-assisted breeding. However, the divergence between wild relatives and cultivated plant species is likely considerable. Consequently, genomic regions of interest in a wild relative may be absent in the corresponding domesticated crop. Additionally, the mapping of DNA sequences present only in wild relatives requires *de novo* assembly rather than resequencing. Many wild crop species genomes have recently been sequenced, and the molecular mechanisms regulating stress resistance have been analyzed and investigated (Bolger *et al.* 2014; Wang *et al.* 2014; Zhang *et al.* 2014; Aversano *et al.* 2015; Wu *et al.* 2016; Xu *et al.* 2017). However, the genomes of wild apple species have not been published, and this lack of genome data inhibits the progress of fruit tree breeding, especially regarding the introduction of unique characteristics from wild species to cultivated varieties (*e.g.*, cold tolerance and disease resistance).

*Malus baccata* is one of four wild apple species that can be freely hybridized with cultivated apple varieties (*Malus domestica*) (Cornille *et al.* 2014). *Malus baccata* is widely used in high-latitude apple-producing areas as a rootstock because of its excellent environmental adaptability and resistance to cold stress (Volk *et al.* 2015). It is also widely distributed throughout China (Figure 1A) in habitats that overlap the apple-producing areas (Liguo and Tao 2003). Thus, *M. baccata* is an important germplasm resource for apple and apple rootstock breeding. We herein describe a high-quality draft genome sequence of the diploid wild apple species, *Malus baccata* (L.) Borkh ‘Shandingzi’. We compared the *M. baccata* genome architecture with that of cultivated apple to screen for genetic signatures of cold tolerance or pathogen resistance. This draft assembly revealed insights into the apple genes underlying cold adaptation and pathogen resistance. The presented data may benefit fundamental research involving the characterization of stress adaptations in fruit trees, but may also be relevant for targeting candidate genes for future breeding programs.

**Figure 1:**
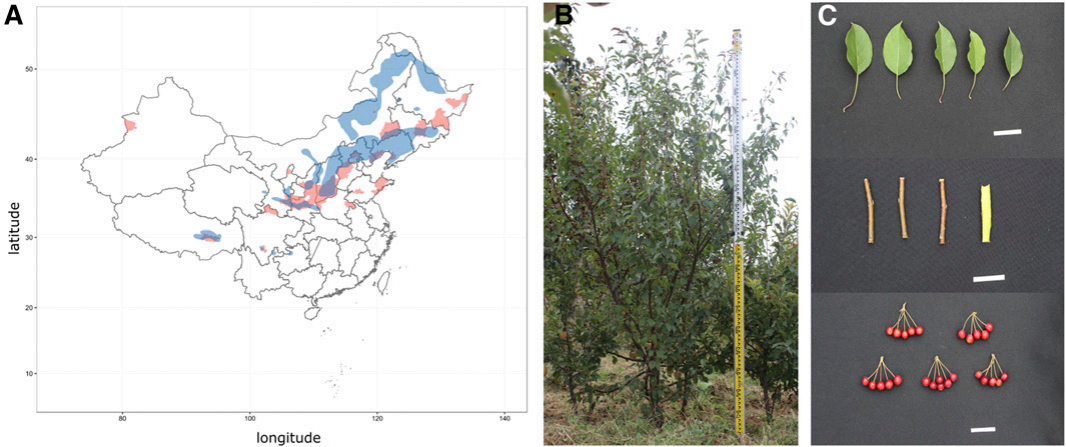
*M. baccata* plant and its distribution. (A) Distribution of apple-producing areas (red) and *M. baccata* natural habitats (blue) in China. (B) *M. baccata* plant. (B) Plant tissues of *M. baccata*. 1, leaf; 2; branch; 3, fruit.

## Materials and Methods

### Plant materials and sequencing

A *Malus baccata* tree from Taigu town, Shanxi province, China (accession No. Y-B094) was used for whole-genome shotgun sequencing. The tree was planted in the Apple Demonstration Nursery of Yangling Modern Agriculture Technology Park (Northwest Agriculture & Forestry University), Shaanxi province, China (34° 52′ N, 108° 7′ E) (Figure 1B and Figure 1C). The root, fruit, phloem, and leaf tissues underwent a transcriptome analysis to assess the annotated genes and evaluate genome quality. All harvested tissues were immediately frozen in liquid nitrogen and stored at −80° until used for DNA or RNA extraction.

Genomic DNA extracted from fresh leaves using a modified CTAB method (Dellaporta *et al.* 1983) was used to construct paired-end libraries. Nineteen paired-end libraries were prepared for sequencing the *M. baccata* genome. These included nine paired-end libraries with an insert of 200, 500, and 800 nt and 10 mate-pair libraries with insert sizes of 2, 5, 10, and 20 kb. All libraries were constructed following the instructions provided by Illumina. After filtering, 173.61 Gb high-quality clean data were retrieved (248-fold genome sequence coverage) (Table S2). Total RNA was isolated from each sample according to an SDS–phenol method (Hu *et al.* 2002). After an agarose gel electrophoresis step, suitable fragments were selected as templates for a polymerase chain reaction (PCR) amplification. During a quality control step, an Agilent 2100 Bioanalyzer and an ABI StepOnePlus Real-Time PCR system were used to quantify and assess the quality of the sample libraries. Finally, the constructed libraries were sequenced with an Illumina HiSeq 2000 system (BGI, Shenzhen, China).

### k-mer analysis of the M. baccata genome

To estimate genome size and heterozygosity, 30.7 Gb high-quality short-insert reads underwent a *k*-mer analysis. The 17-mer frequency distribution derived from the sequencing reads was plotted (Figure S1). The peak for the 17-mer distribution was about 33, and the total *k*-mer count was 25,700,099,451. Thus, the genome size was estimated as 778 Mb (Table S1). A small peak was detected at 1/2 Peak-depth. Therefore, we used simulated heterozygosity rates for the wild apple genome, and conducted 17-mer analyses.

### Genome assembly and evaluation of quality

Genomic DNA isolated from *M. baccata* leaf material was used to construct nine paired-end libraries and 10 mate-pair libraries. All libraries were sequenced using the Illumina HiSeq 2000 sequencing platform and assembled using the SOAPdenovo program (version 2.04.4) (http://soap.genomics.org.cn) (Luo *et al.* 2012). Mate-pair reads were used to construct super scaffolds with the SSPACE program (version 2.0) (Boetzer *et al.* 2011), with sequence gaps filled with GapCloser (version 1.10) (Luo *et al.* 2012).

A high-resolution genetic map with 3,137 SNP markers was created using a mapping population of 390 F_1_ progenies from a cross between *M. baccata* ‘Shandingzi’ and *M. domestica* ‘Danxia’ and restriction site-associated DNA sequencing technology. These markers were analyzed and filtered with Joinmap 4.1 (Ooijen 2006). Genome integrity was assessed using CEGMA (version 2.5) (Parra *et al.* 2007) and BUSCO (version 2.0) (Simão *et al.* 2015).

### Gene and repeat annotations

We used *de novo* and homology-based approaches to analyze the repetitive elements in the *M. baccata* genome. The *de novo* method of annotating repetitive elements involved the RepeatModeler software (Smit and Hubley 2008). Tandem repeats in the genome were annotated with TRF software (version 4.04) (Benson 1999). Transposable elements were identified with a homology-based approach using RepeatMasker and RepeatProteinMask (version 4.0) (http://www.repeatmasker.org/) (Smit *et al.* 2013) as well as the RepBase database (version 16.10) (Jurka *et al.* 2005).

To annotate non-coding RNA, tRNA scan-SE software was used to detect tRNA sequences in the genome according to tRNA structural characteristics. The rRNAs were detected by aligning sequences against known plant rRNA sequences with the BLASTN tool. Using the Rfam family covariance model and Rfam’s INFERNAL software (Griffiths-Jones *et al.* 2005), we predicted the miRNA and snRNA sequence details for the *M. baccata* genome.

We used both homology-based and *de novo* methods to predict genes. Augustus (Stanke *et al.* 2006), GenScan (Burge and Karlin 1997), and glimmerHMM (Majoros *et al.* 2004) were applied for the *de novo* prediction of genes based on a repeat-masked genome. For the homology-based prediction, gene sets from *M. domestica* (Velasco *et al.* 2010), *P. bretschneideri* (Wu *et al.* 2013), *P. persica* (International Peach Genome Initiative *et al.* 2013), *F. vesca* (Shulaev *et al.* 2011), *P. mume* (Zhang *et al.* 2012) and *A. thaliana* (Arabidopsis Genome Initiative 2000), were mapped onto the assembled *M. baccata* genome using TBLASTN (Altschul *et al.* 1990). GeneWise 2.2.0 (Birney *et al.* 2004) was then used to predict gene structures and define gene models based on the complete regions. The complementary gene sets from homology-based and *de novo* predictions were merged to produce a non-redundant reference gene set using GLEAN (http://sourceforge.net/projects/glean-gene/). The RNA-seq data for the four analyzed tissues were also applied to improve the gene annotations. Moreover, the RNA-seq data were mapped to the assembled genome using TopHat (Trapnell *et al.* 2012), and the transcriptome-based gene structures were obtained using Cufflinks (http://cufflinks.cbcb.umd.edu/) (Trapnell *et al.* 2012). The predicted transcripts were used to complement the GLEAN gene set or were integrated as isoforms. We then used the Cuffcompare program (Trapnell *et al.* 2012) to compare the gene set with the previous GLEAN gene set and obtain the final non-redundant gene set.

The proteins encoded in the final non-redundant gene set were functionally annotated according to BLAST searches (E-value cutoff 1 × 10^−5^) of the InterproScan (Zdobnov and Apweiler 2001), SwissProt (Bairoch and Apweiler 2000), and TrEMBL (Bairoch and Apweiler 2000) databases. The pathways enriched among the genes were determined by identifying the best hit in the KEGG database (release 76) (Kanehisa and Goto 2000). We then obtained GO IDs from the corresponding InterPro entries.

### Analysis of genome evolution

The Ka/Ks ratio was calculated with the KaKs_Calculator program (Zhang *et al.* 2006). Gene sets from *M. domestica* (NCBI version)(Velasco *et al.* 2010), *P. bretschneideri* (Wu *et al.* 2013), *P. persica* (International Peach Genome Initiative *et al.* 2013), *F. vesca* (Shulaev *et al.* 2011), *P. mume* (Zhang *et al.* 2012), *A. thaliana* (Arabidopsis Genome Initiative 2000), *C. papaya* (Ming *et al.* 2008), *P. trichocarpa* (Tuskan *et al.* 2006), and *V. vinifera* (Jaillon *et al.* 2007) were used for analyses of genome evolution. Gene clusters, phylogenetic relationships, estimated divergence time, and collinearity and gene family expansion/contraction were analyzed using MCScanX (Wang *et al.* 2012), MrBayes (Huelsenbeck and Ronquist 2001), the MCMCTree program of the PAML package (Yang 2007), and CAFÉ software (De Bie *et al.* 2006), respectively.

### Identification of transcription factors, resistance gene analogs and cold-resistance genes

We searched for consensus transcription factors (TFs) in *M. baccata* using PlantTFDB (http://planttfdb.cbi.pku.edu.cn/) (Jin *et al.* 2014) in HMMER3.0 (http://hmmer.org/). The TFs were classified according to the consensus rules, including the required and prohibited protein domains for each TF gene family summarized on the PlantTFDB website. Accordingly, we predicted TFs for *M. domestica* (Velasco *et al.* 2010), *P. bretschneideri* (Wu *et al.* 2013), *P. persica* (International Peach Genome Initiative *et al.* 2013), and *F. vesca* (Shulaev *et al.* 2011).

We identified resistance gene analogs (RGAs) based on differences in their domains (2015). The TIR, NBS, LRR, and kinase domains were analyzed with hmmsearch in HMMER 3.0, with default thresholds for the Pfam database (http://www.prgdb.org) (Finn *et al.* 2014). The CC, SP, and TM motifs were identified with the paircoil2 program (http://www.cbs.dtu.dk/services/TMHMM/) (McDonnell *et al.* 2006), while SignalP motifs were identified with the SignalP 4.1 Server (http://www.cbs.dtu.dk/services/SignalP/) (Nielsen 2017). We mapped the R gene markers from online resources (http://www.hidras.unimi.it/ and https://www.rosaceae.org/species/malus/all) against the *M. baccata* genome (e-value 1e-5; matched bases ≥ 50 bp; identity ≥ 80%).

To annotate putative cold resistance genes in *M. baccata*, a set of reference proteins were selected from A. thaliana. In detail, 81 proteins annotated with the Gene Ontology term cold acclimation (CA), 40 proteins annotated as cellular response to cold (CRC), and 520 proteins as a response to cold (RC) were selected from TAIR10 GO annotation (https://www.arabidopsis.org/). We analyzed all *M. baccata* protein by BLASTP search using the threshold value as following: e-value < le-30, identity > 50%, query coverage > 80%.

### Data Availability

The genome assembly have been deposited under CNGB Project ID CNA0002537 (https://db.cngb.org/search/project/CNP0000421/). The meta data for the genetic map can be also found in https://db.cngb.org/search/project/CNP0000421/. The genomic raw reads are available via NCBI SRR7248834, SRR7248835, SRR7248837, SRR7248847, SRR7248838 to SRR7248844, SRR7248849 to SRR7248858, SRR7248875 to SRR7248882, and the raw transcriptomic reads are available at NCBI SRR8156047 to SRR8156050. Supplemental material available at FigShare: https://doi.org/10.25387/g3.7523549.

## Results and Discussion

### Genome sequencing and de novo assembly

Our *k*-mer analysis indicated the *M. baccata* genome comprises nearly 779 Mb (Table S1), which was within the flow cytometry data range (709.05–792.18 Mb) (Korban *et al.* 2009). To estimate the heterozygosity of the genome, we approximated the *k*-mer distribution with simulated heterozygous genome sequences, which revealed that the best fit for the real *k*-mer distribution was a simulated *k*-mer distribution (*k* represents the chosen length of substrings) with 1.2% heterozygosity (Figure S1). The scaffolds totaling 719 Mb accounted for approximately 92.32% of the estimated *M. baccata* genome. The result statistics of our final assembly showed that the contig N50 and scaffold N50 values were 44.7 and 452.7 kb, respectively. Additionally, the longest contig and scaffold were 577.9 and 716.2 kb, respectively (Table 1). The *M. baccata* genome GC content is 38.06%, which is very close to that of the *M. domestica* genome (37.99%) (Velasco *et al.* 2010) (Table S3).

**Table 1:** Summary of wild apple (*M. baccata*) genome assembly features

Unit of assembly	Proportion/unit type	No.	Size	% assembly	Length of N50 (kb)	Longest (Mb)
Contigs	All	320,531	665.80Mb	92.6	44.7	0.6
Scaffolds	All	296,545	718.98Mb	100	452.7	7.2
	Anchored	1,561	528.25Mb	73.5		
Repetitive sequences	Total		421.05Mb (58.6%)			
Genes	Total	41,113	126.46Mb (17.6%)			
ncRNA	Total	8,263	1,011.99Kb (0.14%)			

### Construction of a genetic map and establishment of the pseudomolecules

To assemble pseudomolecules, we implemented the genotyping by restriction site-associated DNA sequencing method to construct a high-density *M. baccata* genetic map. We established a high-density genetic map with 3,065 markers using 390 F_1_ progenies from a cross between *M. baccata* ‘Shandingzi’ and *M. domestica* ‘Danxia’. A total of 1,480 scaffolds were anchored to the high-density genetic map by these markers, accounting for 72.57% of the assembly (521.75 of 718.98 Mb). We identified 17 chromosome pseudomolecules and determined the sequence orientation of 53.13% of the anchored scaffolds (382.02 Mb) based on genetic distances (Table S4, Table S5 and Figure S3). Moreover, 39,473 genes located in the anchored pseudochromosomes corresponded to 85.60% of all assembled scaffolds (Table S5). Genetic distance plotted against physical distance revealed that the genetic and physical positions were mostly consistent, except for chr11 (Figure S2).

### Evaluation of assembled genome quality and sequence comparisons

We estimated the completeness of the *M. baccata* genome assembly by attempting to align 325,636 *Malus* species expressed sequence tag (EST) from the GenBank database with the assembly sequence. We observed that 95.22% of the EST were aligned. Because most ESTs were from *M. domestica*, only 84.84% of the EST had least 90% of their lengths covered in the alignments (Table S6). Meanwhile, RNA sequencing (RNA-seq) reads for root, fruit, phloem, and leaf tissues were aligned with the assembly sequence. An average of 95.00% of the read pairs were covered by the assembly sequence for four samples (Table S7). Additionally, we aligned 245 sequences from the nonredundant core eukaryotic genes (CEGs) with the genome assembly using the CEGMA (Core Eukaryotic Genes Mapping Approach) pipeline (Parra *et al.* 2007). A total of 222 (94%) CEG homologs were detected in the *M. baccata* genome (Table S8). We also used the BUSCO (Benchmarking Universal Single-Copy Orthologs) pipeline (Simão *et al.* 2015) to examine the coverage of highly conserved genes to validate the completeness of the *M. baccata* genome assembly. We observed that 93.20% of the plant BUSCO sequences searched were present in *M. baccata* scaffolds (Table S9). The percentage of BUSCOs for the *M. baccata* genome assembly was higher than that for the *M. domestica* (Velasco *et al.* 2010) and *P. bretschneideri* (Wu *et al.* 2013) genomes, but lower than that for the *P. persica* (International Peach Genome Initiative *et al.* 2013) and *F. vesca* (Shulaev *et al.* 2011) genomes (Figure S4). Thus, most of the conserved core gene set was present in the *M. baccata* genome assembly.

### Heterozygosity of Malus Baccata genome

The *M. baccata* genome has high levels of heterozygosity because of self-incompatibility. We mapped the reads with 500-bp inserts onto the draft assembly. A total of 3,759,523 heterozygous single nucleotide polymorphisms (SNPs) were identified, corresponding to 5.2 SNPs per kb. Thus, the heterozygosity was about 0.5%, which was lower than our estimate (1.2%). *M. baccata* heterozygosity rate is similar to the corresponding rates for poplar (about 0.5%) (Tuskan *et al.* 2006), kiwifruit (0.536%) (Huang *et al.* 2013), and orchid (0.4%) (Cai *et al.* 2014), but is lower than the rate for pear (1.02%) (Wu *et al.* 2013), and tea (2.67%) (Xia *et al.* 2017).

We evaluated the structural and functional effects of heterozygous SNPs. The distribution profile revealed that 61.3% of SNPs were within 50 bp of each other, and nearly 25% were within < 10 bp of an adjacent SNP (Figure S5). Most of the identified SNPs (81.74%) were located in intergenic regions. Additionally, 32,895 genes included 18.26% of the SNPs, of which 11,608 genes had a SNP rate of < 1% (Table S10 and Figure S6). Genes with high SNP frequencies (> 3%) were associated with plant–pathogen interactions, protein processing in the endoplasmic reticulum, ascorbate and aldarate metabolism, isoquinoline alkaloid biosynthesis, and various pathways (Table S11).

### Transposable elements and gene model annotation

#### Statistics regarding repetitive DNA:

**Figure 2:**
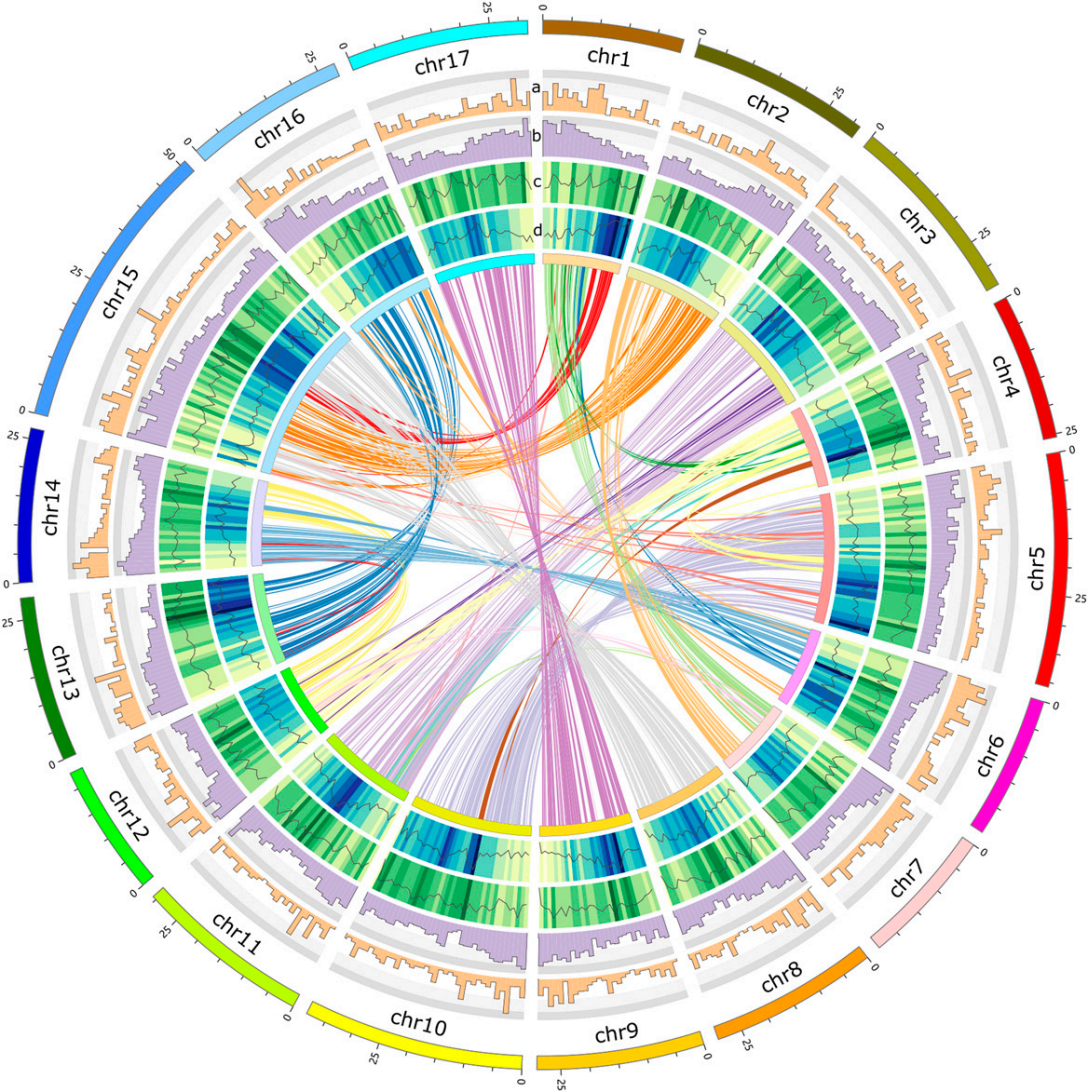
Global view of the *M. baccata* genome. Transcription factor density (track a); gene density (track b); LTR-Gypsy number density (line) and size density (heat map) (track c); LTR-Copia number density (line) and size density (heat map) (track d). The innermost circle represents ideograms of 17 pseudochromosomes and the syntenic relationships of gene blocks from different pseudochromosomes.

Transposable elements (TEs) contributed 58.56% (413.09 Mb) of the *M. baccata* genome sequence (Table S12) which is similar to the annotation rate of *M. domestica* TEs. The long terminal repeat (LTR) retrotransposons were the most abundant transposable elements, representing 44.37% of the assembly. Among the LTR retrotransposons, Gypsy and Copia constituted 29.18% and 16.00% of the *M. baccata* genome sequence, respectively (Figure 2 and Table S13). Other transposable elements included CMC, DNA, hAT, and PIF (Table S14). Moreover, the abundance of LINE/RTE content (8.03%) in the *M. baccata* and *M. domestica* genomes is much greater than that of other Rosaceae plant species (Table S14), suggesting a unique evolutionary event occurred in the *Malus* genome.

#### Protein-coding gene annotation and evaluation:

We conducted RNA-seq experiments for root, phloem, leaf, and fruit libraries to identify genes, ultimately generating a 47.88-Mb transcriptome assembly. Additionally, publicly available *Malus* ESTs and homologs from the sequenced genomes of other Rosaceae species (*i.e.*, *Prunus persica* (International Peach Genome Initiative *et al.* 2013), *Prunus mume* (Zhang *et al.* 2012), *Pyrus bretschneideri* (Wu *et al.* 2013), *M. domestica* (Velasco *et al.* 2010), and *Fragaria vesca* (Shulaev *et al.* 2011)) and from the *Arabidopsis thaliana* genome (Arabidopsis Genome Initiative 2000) were applied for predicting genes. Using evidence-based and *de novo* gene predictions, we identified 46,114 high-confidence protein-coding gene models (Table S15). A similar number of genes was predicted for *M. baccata* (46,114) and *M. domestica* (46,534), while fewer genes were predicted for *P. bretschneideri* (42,269).

The average gene size was 2,667 bp, with a mean of 4.4 exons per gene. The average gene length was similar to that other Rosaceae species such as *P. persica* (International Peach Genome Initiative *et al.* 2013), *P. bretschneideri* (Wu *et al.* 2013) and *F. vesca* (Shulaev *et al.* 2011) (Table S16). Among these genes, 82.01, 33.19, 66.26, and 68.89% were annotated using InterPro (Zdobnov and Apweiler 2001), Gene Ontology (GO) (http://www.geneontology.org/), Kyoto Encyclopedia of Genes and Genomes (KEGG) (Kanehisa and Goto 2000) and Swiss-Prot (Bairoch and Apweiler 2000) databases, respectively, with 39,685 genes (86.06%) annotated by at least one database (Table S17 and Figure S7).

#### Non-coding RNAs:

We also identified and annotated various non-coding RNA sequences in the *M. baccata* genome, including 5,778 ribosomal RNA (rRNA), 1,553 transfer RNA (tRNA), 497 small nuclear RNA (snRNA), and 408 microRNA genes (Table S18).

### Gene family evolution and comparisons

#### Functional annotation of specific genes:

A total of 21,930 (47.56%) *M. baccata* genes exhibited a one-to-one orthology with genes from *M. domestica*. The average Ka/Ks ratio (*i.e.*, ratio of non-synonymous substitutions to synonymous substitutions) for these gene pairs was 0.4770, suggesting that most *M. baccata* genes evolved under purifying selection (Figure S8). A total of 1,574 genes had a Ka/Ks > 1, indicating they may be under positive selection pressure. Additionally, the KEGG pathways enriched among these genes were related to plant hormone signal transduction and plant–pathogen interactions. Furthermore, 25 of these genes were significant at the 0.05 p-value threshold (Table S19). The resulting gene list included four stress resistance genes with a complete structure and three genes with the TIR domain (Table S20).

**Figure 3:**
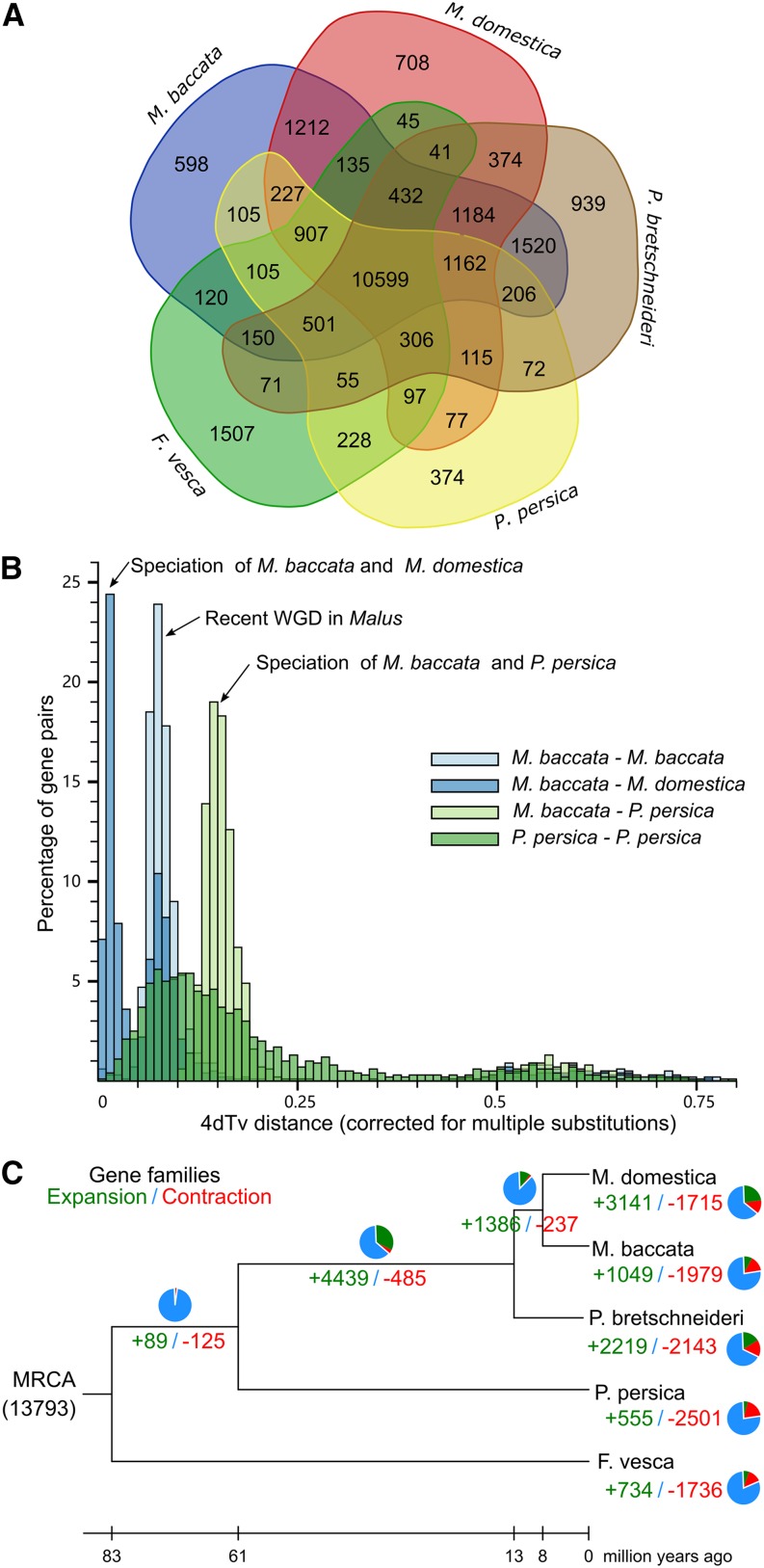
Gene family evolution and comparisons with Rosaceae species, (A) Venn diagram of five Rosaceae species (*M. baccata*, *M. domestica*, *P. bretschneideri*, *P. persica*, and *F. vesca*). (B) Duplications in the *M. baccata* genome revealed *via* 4dTv analyses. (C) Gene family expansions and contractions in five Rosaceae species. Expansions and contractions are indicated in green and red, respectively. The corresponding proportions of the total changes are presented using the same colors in pie charts. The blue sections of the pie charts represent conserved gene families. MRCA, most recent common ancestor.

Comparative analyses involving the *M. baccata* genome and the *M. domestica* (Velasco *et al.* 2010), *P. persica* (International Peach Genome Initiative *et al.* 2013), *P. bretschneideri* (Wu *et al.* 2013) and *F. vesca* (Shulaev *et al.* 2011) genomes revealed that these five Rosaceae species contain a common core set of 10,599 gene families. However, 598 gene families were specific to *M. baccata*, while 708 gene families were specific to *M. domestica* (Figure 3A). The gene families specific to *M. baccata* were enriched in GO terms related to cell division and the cell cycle and KEGG pathways associated with purine metabolism, pyrimidine metabolism, the spliceosome, RNA polymerase, mRNA surveillance, RNA transport, the phagosome, and aminoacyl-tRNA biosynthesis. The identified genes may have contributed to the adaptation of *M. baccata* to environmental conditions. An analysis of the gene families specific to *M. domestica* revealed they were enriched in GO terms and KEGG pathways related to carbohydrate metabolism (*e.g.*, fructose and mannose metabolism and starch and sucrose metabolism).

#### Contraction and expansion of the wild and cultivated apple gene families:

To study gene families that had expanded or contracted only in wild apple or cultivated apple species, we compared the *M. baccata* gene families with those of four other Rosaceae species and ancestral species. We determined that 1,049 and 1,715 gene families had expanded and contracted, respectively, in the *M. baccata* genome relative to its most recent common ancestor (Figure 3C). The results of a KEGG pathway enrichment analysis revealed that the expansion of these families involved genes related to cutin, suberin, and wax biosynthesis as well as fatty acid biosynthesis and degradation (Table S21).

We also examined the pathways associated with the expanded and contracted *M. baccata* and *M. domestica* gene families (Figure 3C). Most of the expansions and contractions were consistent between the two *Malus* species (Table S22). The only differences were that the gene families related to the isoflavonoid biosynthesis pathway and tyrosine metabolism pathway expanded in *M. baccata*, but contracted in *M. domestica*.

#### Phylogenetic analysis:

We estimated the divergence time of 10 sequenced plant species (*i.e.*, *M. baccata*, *M. domestica*, *P. bretschneideri*, *P. persica*, *P. mume*, *F. vesca*, *Carica papaya*, *A. thaliana*, *Populus trichocarpa*, and *Vitis vinifera*) according to known ranges of divergence time as well as a phylogenetic tree. It is likely that *M. baccata* and *M. domestica* diverged from each other approximately 6.9–11.9 million years ago (Figure S9).

To further characterize the divergence between *M. baccata* and *M. domestica*, we measured the transversions at fourfold degenerate sites (4dTv) for orthologous gene pairs among *M. baccata*, *M. domestica*, and peach (Figure 3B). The 4dTv distribution indicated there are two significant groups of blocks, suggesting a recent whole-genome duplication (WGD) event occurred in *M. baccata* and *M. domestica*, but not in peach.

#### Synteny analysis:

An analysis of the synteny between *M. baccata* and other rosaceous species (Table S23) revealed that *M. baccata* and *M. domestica* share similar chromosome structures and organization. All 17 *M. baccata* chromosomes were similar to the corresponding *M. domestica* chromosomes (Figure S10). The self-collinearity of *M. baccata* (Figure 1 and Figure S10) enabled the identification of syntenic chromosome pairs, including LG3 and LG11, LG5 and LG10, LG9 and LG17, and LG13 and LG16, and revealed the chromosomal rearrangements in the *M. domestica* and *P. bretschneideri* genomes.

### Identification of transcription factors and comparison between wild and domesticated apple

Transcription factors (TFs) are important for plant growth and development. We identified 2,345 TF-encoding genes in 58 families in the *M. baccata* genome. These genes represented 5.06% of the genes in the *M. baccata* genome (46,114). The proportion of the genome represented by TF-encoding genes was similar in *M. baccata* and *P. bretschneideri*, but was lower in *M. domestica*. In the *M. baccata* genome, the most abundant TF-encoding genes belonged to the following TF families: bHLH (208 genes), MYB (180 genes), NAC (175 genes), ERF (157 genes), and C2H2 (147 genes) (Table S24). The identification of these TFs may be useful for future functional verifications of *M. baccata* traits.

**Figure 4:**
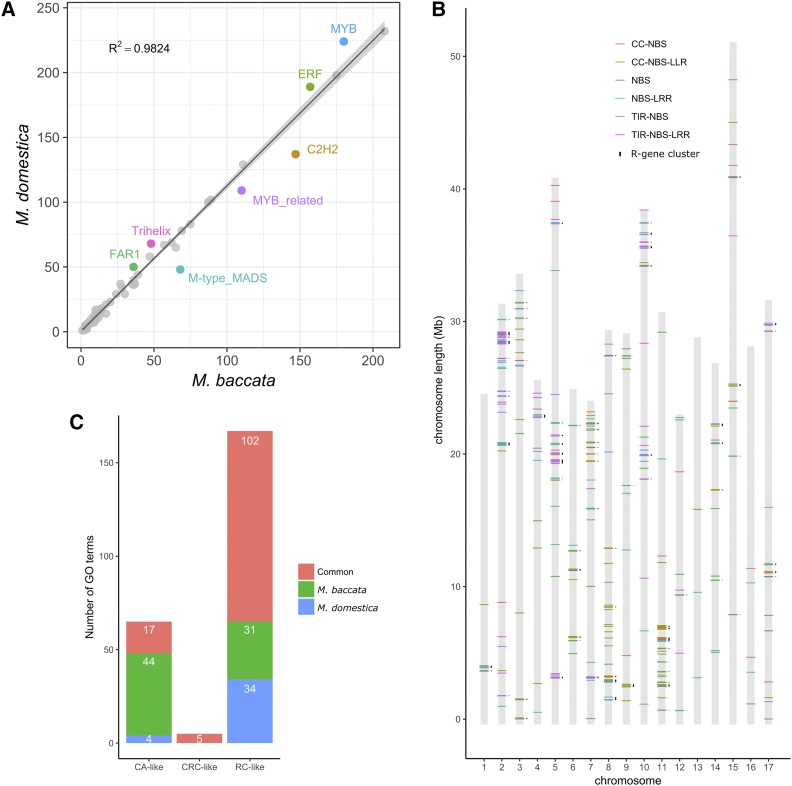
Transcription factor, R gene cluster and cold-responsive genes in *M. baccata* genome. (A) Transcription factor families with more than 50 members in *M. baccata* and *M. domestica*. The confidence interval (*P* = 0.01) of the regression curve is indicated (lines and shadow). Transcription factor families (with more than 50 members) that deviate significantly from the regression curve are indicated. (B) Distribution of *R* gene clusters along *M. baccata* chromosomes. Different colored horizontal bars represent different kinds of NBS *R* genes. The NBS *R* gene clusters are indicated next to the vertical bars representing chromosomes. (C) Results of GO analyses of the *M. baccata* and *M. domestica* COR (cold-responsive) genes. CA, cold acclimation; CRC, cellular response to cold; RC, response to cold.

We compared the abundance of different TF types among Rosaceae genomes (Table S24). A comparison between *M. baccata* and *M. domestica* (Figure 4A) indicated there are fewer MYB and ERF TFs in the *M. baccata* genome than in the *M. domestica* genome. Some MYB family TFs induce fruit coloration, while the ERF family TFs promote fruit growth and ripening. The number of genes encoding these TFs increased in *M. domestica*, which may be related to the domestication of apple trees and their adaptation to the environmental conditions in central Asia. Meanwhile, the *M. baccata* genome was observed to carry more M-type MADS, MYB-related, and C2H2 TFs than the *M. domestica* genome.

### Resistance gene analogs and cold-responsive genes

Disease resistance is the major focus of apple scion and rootstock breeding. The recognition of pathogen effectors is mainly mediated by plant disease-resistance genes, which can be grouped according to encoded motifs [*i.e.*, nucleotide-binding site leucine-rich repeat (NBS-LRR) or transmembrane leucine-rich repeat (TM-LRR)] (Hammond-Kosack and Jones 1997). We compared the identified resistance gene analogs (RGAs) in the *M. baccata* and *M. domestica* genomes, and created a catalog comprising 462 and 800 NBS genes from *M. baccata* and *M. domestica*, respectively. We classified the corresponding genes into various structural categories based on the arrangement of the encoded domains (Table 2). In *M. baccata*, 119 coiled coil-NBS-LRR (CNL), 111 toll/interleukin receptor (TIR)-NBS-LRR (TNL), 126 NBS-LRR, 54 NBS, 23 CC-NBS, and 29 TIR-NBS genes were identified. In contrast, we detected 108 CNL, 153 TNL, 241 NBS-LRR, 136 NBS, 58 CC-NBS, and 104 TIR-NBS genes in *M. domestica*. The huge difference number of NBS R gene between the two genome is that *M. domestica* genome have a lot of single domains or incomplete structures NBS genes. Studies have reported that incomplete structures NBS genes can act as recruiters of or interact with other NBS-LRR proteins (Kohler *et al.* 2008; Lozano *et al.* 2012). Furthermore, the number and diversity of NBS genes containing the RPW8 domain were greater in *M. baccata* than in *M. domestica*; these genes are responsive to powdery mildew infection in many plant species (Table 2). We mapped the NBS genes to 17 pseudochromosomes and observed that they were nonrandomly distributed (Figure 4B). More NBS-LRR R genes were clustered in groups on *M. baccata* chromosomes (66%) than on *M. domestica* chromosomes (61%), and clusters were most abundant on chromosomes 2, 5, and 11 (Table S25). Receptor-like kinase (RLK) protein and receptor-like proteins (RLP) also act as positive regulators in plant innate immunity (Yang *et al.* 2012). And the *M. baccata* genome includes 177 RLK genes and 51 RLP genes, while the *M. domestica* genome carries 93 RLK genes and 74 RLP genes.

**Table 2:** R genes present in the *M. baccata* and *M. domestica* genomes

*R* gene type	*M. baccata*	*M. domestica*
Canonical *R* genes		
CC-NBS-LLR	119	108
TIR-NBS-LRR	111	153
Single domains or incomplete structures		
NBS-LRR	126	241
NBS	54	136
CC-NBS	23	58
TIR-NBS	29	104
Canonical transmembrane domains		
RLK	177	93
RLP	51	74
NBS gene with RPW8 domain		
RPW8-NBS	17	19
RPW8-SBP-NBS	1	0
RPW8-RPW8-RPW8-NBS	1	0
RPW8-NBS-LRR	1	0
RPW8-RPW8-SBP-NBS-HMA	1	1
RPW8-RPW8-SBP-NBS-Pkinase	1	1

The CBF transcription factor along with other genes, can sensing low temperature, initiating the process of cold acclimation and inducing the expression of the cold regulated (COR) genes proteins. And the COR genes can reduce the damage of plant cells due to freeze-induced dryness and the presence of extracellular ice (Miura and Furumoto 2013). A total of 2,978 and 5,089 predicted protein sequences similar to *A. thaliana* cold-responsive proteins were identified in *M. baccata* and *M. domestica*, respectively (Table 3). In *M. baccata*, 408, 51, and 3,519 proteins were homologous to *A. thaliana* sequences annotated with the GO terms ‘cold acclimation’ (hereafter called CA-like), ‘cellular response to cold’ (hereafter called CRC-like), and ‘response to cold’ (hereafter called RC-like), respectively. In *M. domestica*, 466, 58, and 4,565 proteins were CA-like, CRC-like, and RC-like proteins, respectively (Table 3). Enriched GO term categories were detected in both species. For example, *M. baccata* and *M. domestica* CA (associated with cold acclimation) genes were annotated with 44 and 4 unique GO terms, respectively (Figure 4C). Among CBF dependent cold signaling pathways, *CBF* genes induce COR genes expression by binds to DREB motif in COR genes promoter (Sakuma *et al.* 2002). We identified the number of COR genes with DREB motif and observed that the proportion of COR genes that contained the DREB motif was higher for *M. baccata* (50.25%) than for *M. domestica* (42.84%) (Table 3), implying *M. baccata* can induce more cold-responsive biological processes.

**Table 3:** Cold-related (COR) genes and the proportion of COR genes with a DREB motif in the *M. baccata* and *M. domestica* genomes

	*M. baccata*	*M. domestica*
RC gene number	3519	4565
RC gene number with DREB motif	1758	1940
CRC gene number	51	58
CRC gene number with DREB motif	29	29
CA gene number	408	466
CA gene number with DREB motif	212	211
Total COR gene number	3978	5089
Total gene number with DREB motif	1999(50.25%)	2180(42.84%)

CA: Cold Acclimation, CRC: Cellular Response to Cold, RC: Response to Cold.

## Conclusions

We herein describe the first wild apple genome assembly, which was obtained by paired-end sequencing. The assembled genome sequence comprises 665 Mb, with a scaffold N50 value of 452 kb. Future genome-wide comparative studies will provide novel insights into the genomic evolution of Rosaceae species, especially *Malus* species. The annotation of the protein-coding genes and comparisons with the *M. domestica* genome provided insights into *M. baccata*-specific traits, particularly those involved in cold tolerance and pathogen resistance. The analyses of the *M. baccata* genome described herein may be relevant for future investigations of the genetic variations in wild apple germplasm, and may facilitate marker-assisted breeding for apple cultivars and stock exhibiting disease and cold resistance.
